# Tissue Distribution and Elimination of Ciguatoxins in *Tridacna maxima* (*Tridacnidae*, Bivalvia) Fed *Gambierdiscus polynesiensis*

**DOI:** 10.3390/toxins10050189

**Published:** 2018-05-10

**Authors:** Mélanie Roué, Hélène Taiana Darius, André Ung, Jérôme Viallon, Manoella Sibat, Philipp Hess, Zouher Amzil, Mireille Chinain

**Affiliations:** 1 Institut de Recherche pour le Développement (IRD)—UMR 241 EIO, PO box 53267, 98716 Pirae, Tahiti, French Polynesia; 2 Institut Louis Malardé (ILM), Laboratory of Toxic Microalgae—UMR 241-EIO, PO Box 30, 98713 Papeete, Tahiti, French Polynesia; tdarius@ilm.pf (H.T.D.); aung@ilm.pf (A.U.); jviallon@ilm.pf (J.V.); mchinain@ilm.pf (M.C.); 3 IFREMER, Phycotoxins Laboratory, F-44311 Nantes CEDEX, France; manoella.sibat@ifremer.fr (M.S.); philipp.hess@ifremer.fr (P.H.); zouher.amzil@ifremer.fr (Z.A.)

**Keywords:** giant clams, ex situ exposure to toxic algae, ciguatoxins, *Gambierdiscus polynesiensis*, anatomical distribution, toxin elimination, CBA-N2a, LC-MS/MS

## Abstract

Ciguatera is a foodborne disease caused by the consumption of seafood contaminated with ciguatoxins (CTXs). Ciguatera-like poisoning events involving giant clams (*Tridacna maxima*) are reported occasionally from Pacific islands communities. The present study aimed at providing insights into CTXs tissue distribution and detoxification rate in giant clams exposed to toxic cells of *Gambierdiscus polynesiensis*, in the framework of seafood safety assessment. In a first experiment, three groups of tissue (viscera, flesh and mantle) were dissected from exposed individuals, and analyzed for their toxicity using the neuroblastoma cell-based assay (CBA-N2a) and liquid chromatography-tandem mass spectrometry (LC-MS/MS) analyses. The viscera, flesh, and mantle were shown to retain 65%, 25%, and 10% of the total toxin burden, respectively. All tissues reached levels above the safety limit recommended for human consumption, suggesting that evisceration alone, a practice widely used among local populations, is not enough to ensure seafood safety. In a second experiment, the toxin content in contaminated giant clams was followed at different time points (0, 2, 4, and 6 days post-exposure). Observations suggest that no toxin elimination is visible in *T. maxima* throughout 6 days of detoxification.

## 1. Introduction

Pacific Island Countries and Territories (PICTs) communities are strongly dependent on marine products for their subsistence and income [1,2]. Thus, seafood contamination by marine biotoxins represents a serious impediment to the economy of PICTs populations. Although fish are the most widely consumed marine products in PICTs, several species of marine invertebrates, including giant clams, are also part of the diet of local populations. As an example, reported catches of giant clam meat reached tens of tons annually during the 1970s and 1980s in Tonga, Fiji, and French Polynesia [3]. While similar levels of exploitation were still reported in the early 2000s from small and isolated islands of French Polynesia [4], the local market of giant clam meat from the East Tuamotu and Australes archipelagos now exceeds 70 tons per year [5]. Giant clams grow in quite shallow depths, so are easily harvested by hand. Even though some people still consume it, the hepatopancreas is most often eviscerated before consumption in local communities, probably due to its visual appearance and bitter taste, but also as a precaution (in the same way as the liver of fish is typically removed).

Ciguatera fish poisoning (CFP) is a seafood-borne illness classically caused by the consumption of tropical coral reef fish contaminated with ciguatoxins (CTXs), polyether neurotoxins produced by dinoflagellates of the genus *Gambierdiscus* [6]. The clinical manifestations of the disease classically involve gastrointestinal disorders, cold allodynia, itching, paresthesia, asthenia, muscular pain, and dizziness, together with other neurological and cardio-vascular symptoms [7]. Although coral reef fish are usually regarded as the primary vectors of ciguatera, atypical ciguatera-like poisoning events following the consumption of giant clams (*Tridacna maxima*), marine gastropods (*Tectus niloticus*), and sea urchins (*Tripneustes gratilla*) are also occasionally reported from PICTs, most notably from French Polynesia, New Caledonia, Cook Islands, and the Republic of Vanuatu [8,9,10,11,12,13,14]. Besides the symptoms typical of CFP, atypical signs were also observed, such as the rapid onset of the disease (occurring within minutes), alteration of taste, burning sensation on the tongue and throat, as well as paralysis. In the case of poisoning events involving *T. maxima*, toxicological analyses using the receptor binding assay (RBA) and the neuroblastoma cell-based assay (CBA-N2a) confirmed the presence of liposoluble compounds acting on voltage-gated sodium channels (VGSCs). These toxins also displayed a mode of action and chemical properties very similar to those of CTXs [11,15,16]. Unfortunately, confirmation of the exact nature of these compounds could not be achieved from wild samples.

Recently, Roué et al. (2016) [17] provided confirmation that giant clams should be regarded as potential vectors of ciguatera, particularly in fishing areas frequently exposed to *Gambierdiscus* blooms. These authors showed that *T. maxima* fed highly toxic cells of *Gambierdiscus polynesiensis* were able to bio-accumulate algal CTXs, at concentrations well above the safety limit commonly recommended for human consumption in the Pacific region. However, since toxin analyses were conducted on extracts prepared from whole specimens, it was not possible to tell whether CTXs were differentially concentrated in the tissues of contaminated animals. Likewise, the study by Roué et al. (2016) [17] did not provide any information about the detoxification time for CTXs in giant clams. Indeed, such information has significant public health implications and may help identify practical measures to limit the risk of poisoning among consumers, e.g., by simply removing the contaminated tissues and/or issuing a quarantine period within distribution channels whenever necessary.

In this context, two additional series of ex situ exposures to toxic *Gambierdiscus* cells were conducted to address the following issues: (i) assess the differential tissue distribution of CTXs in contaminated individuals; and (ii) assess whether any elimination of CTXs is visible from contaminated giant clams over a short-term period of one week, assuming that these bivalves belong to the group of “rapid detoxifiers” [18]. To achieve this, giant clams were exposed to in vitro cultures of the highly toxic strain TB92—*G. polynesiensis* in two independent experiments. In the first experiment (“tissue distribution study”), the exposed specimens were dissected into three tissues: viscera, mantle and flesh (i.e., rest of the body tissues, including gills) which were further tested for their toxicity. In the second experiment (“detoxification study”), contaminated individuals were sacrificed at different time intervals (0, 2, 4, and 6 days) following their transfer into clean water, and tested for their toxicity. Detection of CTXs in samples was achieved using the CBA-N2a and liquid chromatography—tandem mass spectrometry (LC-MS/MS) analyses.

## 2. Results

### 2.1. Tissue Distribution Study

Giant clams (*n* = 3 per tank) were placed in four independent tanks and exposed to *G. polynesiensis* cells (tank No. 1, 2, and 3) or kept in clean seawater (control tank). After two days of exposure, giant clams were sacrificed and dissected into three tissues: viscera (consisting mainly of the hepatopancreas), mantle and flesh (i.e., rest of the body tissues, including gills). Then, each group of tissues was pooled for each tank before further analyses.

Extracts obtained from viscera, mantle and flesh of control giant clams showed no toxicity as confirmed by CBA-N2a and LC-MS/MS analyses (data not shown).

**Figure 1:**
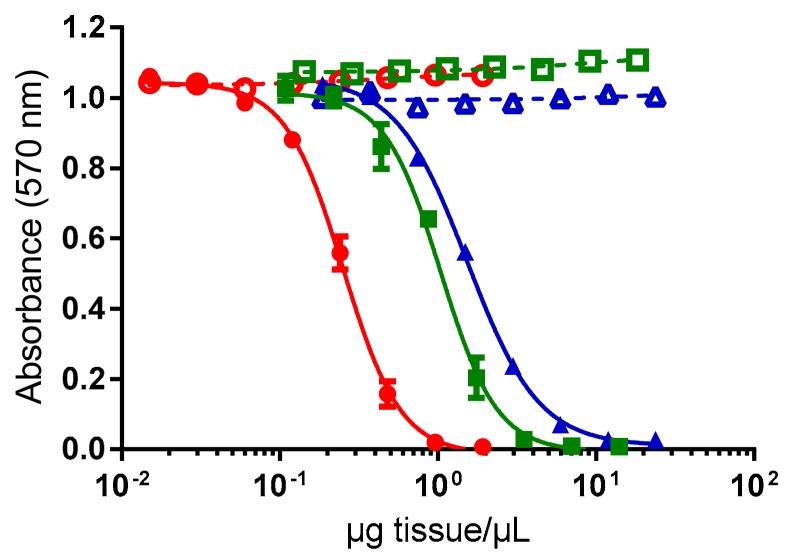
Dose-response curves of Neuro-2a cells in absence (open symbols) and presence (solid symbols) of ouabain and veratridine (OV^−^ and OV^+^ conditions, respectively), when exposed to different concentrations of extracts obtained from viscera (○/●), flesh (□/■) and mantle (△/▲) of giant clams exposed to *G. polynesiensis* cells (TB92 strain). Data represent the mean ± SD of the three experimental tanks, each tissue tested in three independent CBA-N2a experiments (each run in triplicates).

Conversely, all extracts corresponding to viscera (*n* = 3), mantle (*n* = 3) and flesh (*n* = 3) of exposed giant clams were found toxic using CBA-N2a. As shown in Figure 1, corresponding to the mean of the three tanks for each tissue, a sigmoidal dose-response curve was obtained in OV^+^ conditions (i.e., in the presence of ouabain and veratridine) whereas no cytotoxic effects were observed on Neuro-2a cells in OV^−^ conditions (i.e., in absence of ouabain and veratridine), a response typical of the presence of CTXs in these samples. The mean EC_50_ values (*n* = 3 tanks) were 0.24 ± 0.02, 1.21 ± 0.29, and 1.65 ± 0.52 µg tissue/µL for viscera, flesh and mantle, respectively (Figure 1).

Absolute toxicities of tissues (i.e., toxin contents in each tissue) from giant clams from each of the three tanks indicated that viscera was 4- to 6-fold more toxic than flesh and 4- to 9-fold more toxic than mantle (Table 1), with mean absolute toxicities (*n* = 3 tanks) of 13.0 ± 1.1, 2.7 ± 0.6, and 2.0 ± 0.6 ng P-CTX3C equiv./g tissue, respectively.

**Figure 2:**
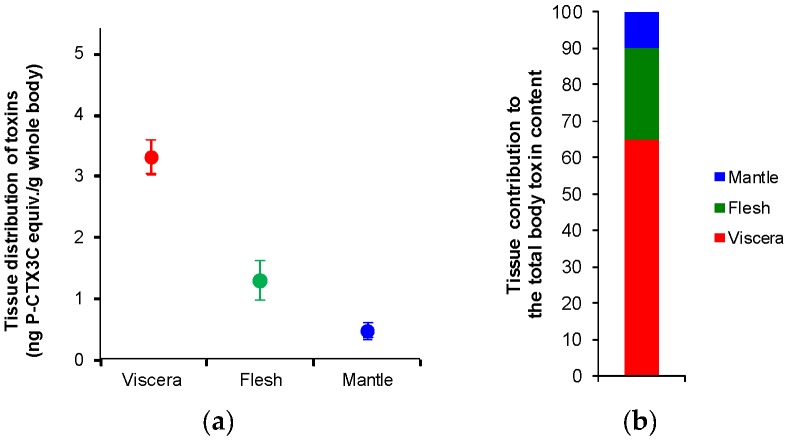
(**a**) Tissue distribution of toxins and (**b**) tissue contribution to the total body toxin content in giant clams exposed to *G. polynesiensis* cells (TB92 strain), as assessed by CBA-N2a data. Data represent the mean ± SD of the three experimental tanks, each tissue tested in three independent CBA-N2a experiments (each run in triplicates).

Regarding the relative weight contribution of each tissue to the whole body weight of giant clams, flesh was the most abundant tissue (around 50%), followed by mantle and viscera (around 25% each) (Table 1). The tissue distribution of toxins (as expressed per g of whole body) was then calculated as a function of absolute toxicity and relative weight contribution (Table 1). For each of the three tanks, despite the limited contribution of viscera to the total body mass of giant clams, the toxin distribution in this tissue was 2- to 3-fold and 5- to 8-fold higher than what is observed in the flesh and the mantle, respectively (Table 1). With a mean toxin distribution (*n* = 3 tanks) of 3.3 ± 0.3, 1.3 ± 0.3 and 0.5 ± 0.1 ng P-CTX3C equiv./g whole body for viscera, flesh and mantle, respectively (Figure 2a), the contribution of viscera to the whole toxin content averaged 65%, followed by the flesh and the mantle with 25% and 10%, respectively (Figure 2b).

**Figure 3:**
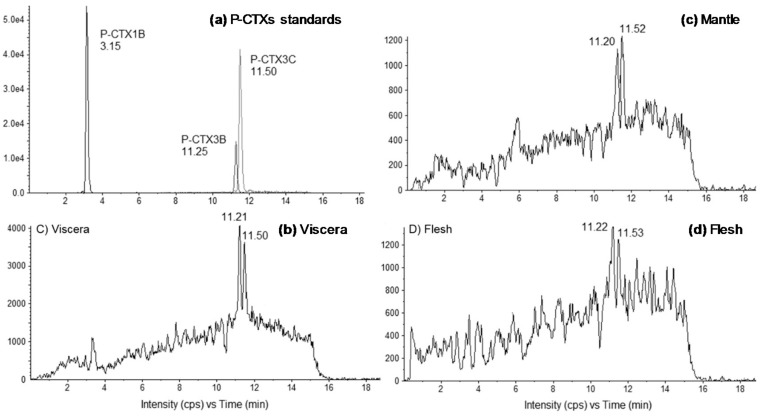
Liquid chromatography-tandem mass spectrometry (LC-MS/MS) chromatograms of (**a**) P-CTXs standards, and fractions obtained from (**b**) viscera, (**c**) mantle and (**d**) flesh of giant clams exposed to *G. polynesiensis* cells (TB92 strain). Chromatograms were acquired following the procedure described in Section 4.5, in positive multi-reaction monitoring mode. For (**d**), S/N < 3.

The presence of CTXs in toxic fractions was further confirmed by LC-MS/MS analyses, with P-CTX3B (RT 11.2 min) and P-CTX3C (RT 11.5 min) present in similar proportions (≈1:1) in all tissues (i.e., mantle, viscera or flesh) (Figure 3). The toxin content of viscera was higher than in the flesh and the mantle (where CTXs were detected at the limit of quantification) (Figure 3), confirming that viscera was the main storage tissue for CTXs compared to the other two tissue types. It should be noted that LC-MS/MS data in this study are mostly semi-quantitative as concentrations were low and no certified standards exist for any of the ciguatoxins.

### 2.2. Detoxification Study

As in the first experiment, giant clams (*n* = 3 per tank) were placed in five independent tanks and exposed to *G. polynesiensis* cells (*n* = 4 tanks) or kept in clean seawater (control tank). After two days of exposure to *G. polynesiensis* cells, the seawater was renewed for the detoxification study. Each exposed tank was then randomly assigned a time point (0, 2, 4, and 6 days post-exposure) at which point the giant clams were sacrificed. After sacrifice, each whole animal was independently analyzed for its toxicity using CBA-N2a.

**Figure 4:**
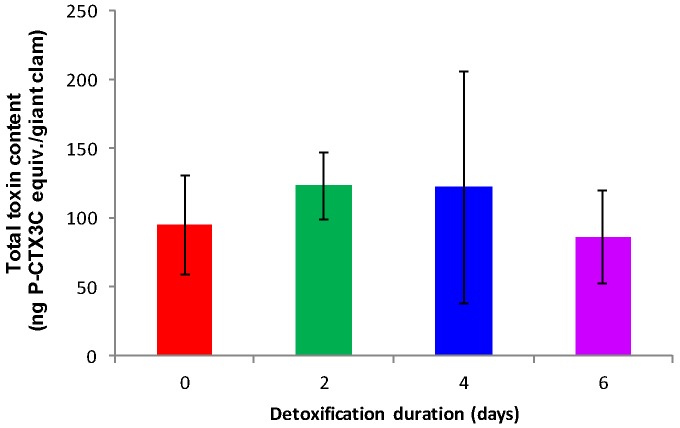
Mean total toxin burdens in contaminated giant clams, as assessed by CBA-N2a, at 0, 2, 4, and 6 days post-exposure. Data represent the mean ± SD of three individual giant clams, each of them tested in three independent CBA-N2a experiments (each run in triplicates).

Fractions obtained from control animals showed no toxicity using CBA-N2a (data not shown). Conversely, all fractions obtained from contaminated giant clams after 0 (*n* = 3), 2 (*n* = 3), 4 (*n* = 3), and 6 (*n* = 3) days of detoxification were found toxic, with a sigmoidal dose-response curve typical of the presence of CTXs (data not shown). The mean toxin contents (*n* = 3 giant clams) were 1.8 ± 0.4, 2.5 ± 0.7, 2.0 ± 1.1, and 2.1 ± 0.7 ng P-CTX3C equiv./g whole body for 0, 2, 4, and 6 days of detoxification, respectively (Table 2). Thus, no significant elimination of toxins had occurred (ANOVA, *p* > 0.05) and whole giant clams contained a mean of 114 ± 54 ng P-CTX-3C equiv./giant clam all along the course of the detoxification period (Figure 4).

Each tank of three giant clams exposed to *G. polynesiensis* cells received a total toxin load of 17.25 µg P-CTX3C equiv. As shown in Figure 4, at the end of the exposure period (i.e., 0 days of detoxification), each giant clam had accumulated around 95 ng P-CTX3C equiv., consequently, it was estimated that each giant clam had retained only 0.6% of the total toxin load administered in each tank. This percentage stayed stable throughout the full detoxification experiment.

## 3. Discussion

### 3.1. Anatomical Distribution of CTXs in Toxic Giant Clams

Results of CBA-N2a analyses indicated that all the tissues (i.e., viscera, mantle, and rest of the flesh) of giant clams fed toxic *G. polynesiensis* cells, were able to retain CTXs. These findings confirmed previous observations on the ability of giant clams to bio-accumulate CTXs in their tissues [17]. In the present study, CTXs were preferentially concentrated in the viscera (mostly composed by the hepatopancreas), which was 2.5 and 6.7-fold more toxic than the flesh (i.e., the rest of body tissues, including gills) and mantle, respectively. This preferential accumulation of toxins in the viscera is consistent with a digestive uptake route of CTXs in this bivalve mollusk, as previously hypothesized by Roué et al. (2016) [17]. Indeed, following the filtration and ingestion of *G. polynesiensis* cells by *T. maxima*, the subsequent distribution of CTXs to non-visceral tissues could well explain the lower toxicities observed in the flesh and mantle. This differential accumulation of toxins in *T. maxima* tissues is coherent with the chemical nature of CTXs since lipophilic toxins are often concentrated in digestive glands; for instance, CTXs are known to be preferentially accumulated in the liver of fish [19,20]. Our results are also in agreement with previous studies conducted on other bivalve species, which concluded that in filter-feeding marine organisms exposed to harmful algal blooms, most of the toxin uptake accumulates in the viscera (most notably the digestive gland) despite the limited contribution of this tissue to the animal total body mass [18,21,22,23,24,25,26,27,28,29,30]. As an example, in the Japanese scallop *Patinopecten yessoensis* exposed to cultured *Dinophysis fortii* cells, dinophysistoxins (DTXs) and pectenotoxins (PTXs) were almost exclusively found in the digestive gland with only low levels being detected in the gill, mantle, gonad, and adductor muscle [27]. However, the preferential accumulation of toxins in non-digestive and non-visceral tissues has also been documented in a limited number of studies [28,31,32,33]. For example, the highest concentrations of okadaic acid-group (OA) toxins were detected in the adductor muscle of *M. chilensis* collected in areas where *Alexandrium catenella*, *Dinophysis acuminata* and *Dinophysis acuta* cells were observed [28]. Moreover, even if up to 90% of the azaspiracid (AZA) burden was found in digestive gland of mussels exposed to *Azadinium spinosum* cells, Jauffrais et al. (2012) [30] also showed that different algal densities or dissolved toxins may contribute to accumulation via different mechanisms, e.g., direct accumulation in gills or other tissues. Thus, the fact that up to approximately 40% of the total body burden was found in non-visceral tissues in this study may indicate that direct accumulation in mantle may also take place in giant clams.

In terms of public health implications, the distribution of toxins found in the viscera of toxic giant clams was 167-fold higher than the safety limit commonly recommended for human consumption, i.e., 0.01 ng P-CTX1B/g or 0.02 ng P-CTX3C/g [34,35]. Likewise, the flesh and mantle in toxic individuals also contained toxin levels well above this safety limit, i.e., 66 and 25-fold higher, respectively. Such observations clearly show that the common practice of removing the animal hepatopancreas before consumption of giant clams, will certainly contribute to reduce the amount of toxins ingested, but does not guarantee an effective protection of consumers as ca. 30–40% of the total toxin burden remains in non-visceral tissues. Similar conclusions apply to two bivalve mollusks, *V. antiqua* and *G. solida,* for which only the foot is consumed. Indeed, in their study, García et al. (2015) [28] showed that in addition to the digestive gland, foot also accumulates significant levels of toxins in these two species, and could thus be highly harmful to consumers. In contrast, the amount of DTXs detected in the adductor muscle of the Japanese scallops *P. yessoensis* was extremely low, confirming the validity of the practice of evisceration implemented in Japan since 1980 [27]. Still, poor dissection practices may also lead to contamination of non-visceral tissues as shown for domoic acid in scallops [36] and therefore, great care should be taken with evisceration as a means of toxin-reduction.

The TB92—*G. polynesiensis* strain is known to produce multiple P-CTX congeners, i.e., P-CTX3B, P-CTX3C, P-CTX3C/3B analogs, P-CTX4A, P-CTX4B, and M-seco-P-CTX4A [37], as confirmed by LC-MS/MS analyses performed on *Gambierdiscus* cultures used for exposure experiments [17]. Here, LC-MS/MS data indicated that P-CTX3B and P-CTX3C, present in almost similar proportions (assuming the same response factor in LC-MS/MS for P-CTX3B and P-CTX3C), were the only known analogs detected in all tissue samples, even though the potential presence of other CTX congeners at concentrations below the limit of detection of the technique cannot be completely ruled out. Interestingly, P-CTX3B and P-CTX3C were also among the major congeners detected in *Tectus niloticus* (Gastropod) and *Tripneustes gratilla* (Echinoid) specimens involved in CFP poisoning events in French Polynesia [13,14], suggesting that both congeners are preferentially bio-accumulated by marine invertebrates.

### 3.2. Elimination of CTXs from T. maxima

Results of CBA-N2a analyses showed that following the transfer of contaminated giant clams into clean seawater, the amount of toxins detected in *T. maxima* at 0, 2, 4, and 6 days post-exposure did not differ significantly. Moreover, giant clams were still not edible after one week in clean seawater (with a toxin content 105-fold higher than the safety limit commonly recommended for human consumption), suggesting that the time lime for toxin detoxification below the safety limit commonly recommended for human consumption in giant clams may take weeks or even months. These results are consistent with field studies conducted on other marine invertebrates species also involved in CFP poisoning events in French Polynesia, which indicated that *Tectus niloticus* (Gastropod) and *Tripneustes gratilla* (Echinoid) have a slow detoxification rate for CTXs. Indeed, based on CBA-N2a results, a 19-fold and 64-fold decrease in the overall ciguatoxicity of *Tectus niloticus* and *Tripneustes gratilla* samples, respectively, was observed over a two-year period, but toxin concentrations were still consistently above the safety limit recommended for human consumption [13,14]. Of note, the authors also underlined that these findings may be confounded by the additional accumulation of CTXs over the study period, even in the presence of low cell numbers of *Gambierdiscus*.

Elimination of toxins is mainly affected by their chemical properties. In general, lipophilic toxins are retained longer than the hydrophilic toxins. In bivalve mollusks, this elimination rate has been shown to also vary according to the species involved, taking from days up to several months, depending on initial concentration and environmental conditions [38]. Classically, bivalve species fall into two general categories in terms of their detoxification capacity: (i) rapid to moderate detoxifiers which eliminate toxins in a few days; and (ii) slow detoxifiers which need several months or even years to eliminate toxins [18,31,33,39,40,41]. For example, the blue mussel *Mytilus edulis* eliminate PSTs in less than 10 days whereas the butter clam *Saxidomus giganteus* need one to four months to depurate the same toxins [18]. In the same way, the clam *Perna viridis* showed a detoxification time for brevetoxins (PbTxs) significantly higher (above 4–5 months) than the oyster *Crassostrea virginica* and the clam *Mercenaria* after a bloom of *Karenia brevis* [41]. Finally, DA detoxification time has been shown to be species-specific and to have a wide-ranging variability, since most mussels depurate DA very quickly [39] whereas the razor clam *Siliqua patula* or the scallop *P. maximus* have much slower detoxification kinetics [31,40]. Results in the present study suggest that *T. maxima* falls into the slow detoxifiers’ category since no significant toxin elimination was visible throughout one week of detoxification; however, further experiments with longer detoxification period will be necessary to firmly conclude this. Furthermore, as for other marine invertebrates [13,14], it would be interesting to study bioaccumulation and elimination rates of CTXs in wild giant clams.

At the end of the exposure period, each giant clam had retained only 0.6% of the toxin burden administered to each tank, corresponding to a total accumulation of 1.8% for each tank containing three giant clams, a result coherent with the study by Roué et al. (2016) [17] which showed an accumulation of around 3% of the toxins in the same experimental conditions. This low toxin uptake rate observed in giant clams is not surprising. For example, a study has shown that the absorption efficiency of DTX1 by the digestive gland of *P. yessoensis* was estimated at less than 3% of the total amount of DTX1 given to the scallops that were fed *D. fortii* cells [42]. Likewise, the bay scallops *Argopecten irradians* exposed to cultured cells of *Prorocentrum lima* showed a toxin assimilation efficiency in their tissues of less than 1% [43]. Interestingly, a biphasic detoxification kinetic is often observed in bivalves, consisting in an initial, more rapid, detoxification phase and a subsequent slower phase of toxin elimination [18,25,30,44,45]. It has been suggested that the initial rapid loss of toxin corresponds to clearance of unassimilated toxins from undigested cells or in dissolved form in the gut lumen, whereas the second slower phase represents the release of assimilated toxins either bound to the digestive gland or incorporated within other tissues [21,25,30,46,47]. In the present study, one hypothesis could be that giant clams could also have a biphasic detoxification kinetic, and that the rapid detoxification of unassimilated CTXs in contaminated animals actually occurred during the exposure period, and that these latter were in the slow phase of toxin elimination during the course of the detoxification study. Furthermore, another hypothesis could be that this low toxin uptake is in fact due to a low ingestion of *Gambierdiscus* cells by giant clams. In any case, further experiments with a longer exposure time may be necessary to better reflect conditions in the natural environment, and should be followed by a longer detoxification period and search for toxins in feces and system circulating-water as well as quantification of remaining *Gambierdiscus* cells in experimental tanks, in order to clarify actual accumulation and detoxification rates in giant clams.

## 4. Materials and Methods

### 4.1. Biological Material

#### 4.1.1. Giant Clams

Giant clams (*T. maxima*) used in this study were purchased from an aquaculture farm in Tahiti (French Polynesia). For the tissue distribution study, giant clams (*n* = 12) were 15.3 ± 1.1 cm long, had a mean shell height of 8.4 ± 1.4 cm and provided a mean flesh wet weight of 60.6 ± 16.6 g. For the detoxification study, giant clams (*n* = 15) were 17.1 ± 1.6 cm long, had a mean shell height of 9.9 ± 1.4 cm and gave a mean flesh wet weight of 54.4 ± 14.2 g.

#### 4.1.2. Cultures of *Gambierdiscus polynesiensis*

A highly toxic strain (TB92—*G. polynesiensis*) available from the algal collection of Institut Louis Malardé was used for the ex situ exposure experiments. TB92 cultures were obtained as previously described in Roué et al. (2016) [17]. Cultures were harvested in their late exponential/early stationary growth phase (i.e., 28-days post-inoculation) when cells exhibit highest CTX levels corresponding to an average toxicity of 5.83 ± 0.85 pg P-CTX3C equiv./cell.

### 4.2. Ex Situ Exposure Experiments

The experimental set-up was similar to the one previously described in Roué et al. (2016) [17]. Experiments were conducted in a closed environment, in tanks containing 20 L of seawater with a salinity value of 37. The temperature and the percentage of dissolved oxygen were stabilized at around 28.8 °C and 8 mg/L, respectively, and controlled daily. The light regime followed a 12:12 h (light:dark) photoperiod with an average irradiance of about 50 µmol photons/m/s of light (daylight fluorescent tubes). Each tank was equipped with a pump set at a flow rate of 200 L/h, in order to favor the suspension and/or dissemination of *Gambierdiscus* cells in the surrounding environment of giant clams. Three giant clams were placed in each experimental tank and acclimated to laboratory conditions during three days prior to exposure. Two independent series of experiments were conducted, in order to assess both the distribution of CTXs in three tissue types and the detoxification rate of CTXs from giant clams, respectively.

#### 4.2.1. Tissue Distribution Study

In three tanks, a cell dose of 150,000 cells was administered 20 times to giant clams over a period of 48 h, to reach a total of 3 × 10^6^ *Gambierdiscus* cells per tank, corresponding to an overall toxin load of 17.25 µg P-CTX3C equiv., which is representative of what can be found in natural blooms, as previously discussed in Roué et al. (2016) [17]. A fourth tank containing animals kept in the same laboratory conditions as exposed animals served as control. The water was not replaced in the course of the exposure period. No mortality was observed during the acclimation step, nor in the course of the exposure experiment.

At the end of the exposure period, each individual was collected and immediately sacrificed. The whole meat was removed from the shell and thoroughly rinsed twice in 0.5 L seawater to remove all trace of incubation water or intervalvar liquid potentially contaminated with *Gambierdiscus* cells and/or dissolved CTXs. Each giant clam sample was further dissected into three tissues: viscera (consisting mainly of the hepatopancreas), mantle and flesh (i.e., rest of the body tissues, including gills). Each group of tissue corresponding to the three giant clams from a same experimental tank was then pooled, ground and stored at −20 °C.

#### 4.2.2. Detoxification Study

Four tanks received each a total cell load of 3 × 10^6^ cells throughout a 48 h exposure period, corresponding to an overall toxin load of 17.25 µg P-CTX3C equiv., in the same conditions that for the tissue distribution study. A fifth tank containing animals maintained in the same laboratory conditions as exposed animals served as control. Following toxic exposure, seawater in each of the five tanks was entirely renewed by clean seawater in order to remove any remaining *Gambierdiscus* cells (data not available) and/or dissolved CTXs. In order, to assess whether any elimination of CTXs is visible from contaminated giant clams over a short-term period of one week, assuming that these bivalves belong to the group of “rapid detoxifiers” [18], each exposed tank was then randomly assigned a time point (0, 2, 4, and 6 days post-exposure) at which to sacrifice the three individuals from the respective tank. For each animal, the whole meat was removed from the shell and thoroughly rinsed twice in 0.5 L seawater. Each sample was then ground separately and stored at −20 °C.

### 4.3. Toxin Extration

Samples were extracted according to the procedure previously described in Roué et al. (2016) [17]. Briefly, 10 g of a given tissue (for tissue distribution study) or 10 g of the whole giant clam body (for detoxification study) were extracted in pure methanol (MeOH), followed by 50% aqueous MeOH. The resulting dried extract was partitioned between dichloromethane (CH_2_Cl_2_) and 60% aqueous MeOH. The CH_2_Cl_2_ phase, likely to contain CTXs, was dried under vacuum and defatted by a second solvent partition using cyclohexane and aqueous MeOH. The methanolic fraction was recovered and further purified using C_18_ Sep-Pak cartridges (360 mg, Waters^®^). The resulting 90% aqueous methanol fraction, likely to contain the majority of CTXs, was dried in a SpeedVac concentrator and stored at +4 °C until CBA-N2a and LC-MS/MS analyses.

### 4.4. Neuroblastoma Cell-Based Assay (CBA-N2a)

CBA-N2a analyses were conducted following the procedure previously described in Roué et al. (2016) [17] as adapted from Manger et al. (1993) [48]. All dry fractions were carefully weighed and re-suspended in methanol to reach a final concentration of 1 mg of dry extract/100 µL prior to CBA-N2a assays. Fractions were tested at a concentration range of 5–9524 pg/µL in eight distinct concentrations and three independent experiments (each run in triplicate). Absorbance data were fitted to a sigmoidal dose-response curve (variable slope) based on the four-parameter logistic model (4PL) allowing the calculation of EC_50_ values using Prism v6.0.7 software (GraphPad, San Diego, CA, USA). The half maximal effective concentration values (EC_50_) ± standard deviations (SD) were expressed in g tissue equiv./µL or g whole body equiv./µL for tissue distribution and detoxification studies, respectively. Toxin contents (*T*) were then estimated using the following formula *T* = (P-CTX3C EC_50_/sample EC_50_) and expressed in ng P-CTX3C equiv./g tissue or ng P-CTX3C equiv./g whole body for tissue distribution and detoxification studies, respectively. According to Bricelj and Shumway (1998) [18], the contribution of a tissue to the total toxin body burden is a function of both its absolute toxicity and relative weight contribution. Thus, the tissue distribution of toxins, expressed in ng P-CTX3C equiv./g whole body, were calculated for each tissue (viscera, mantle, and flesh) using the following formula: absolute toxicity (expressed in ng P-CTX3C equiv./g tissue) × relative weight contribution (in %). The EC_50_ value obtained for P-CTX3C, the maximum concentration of dry extracts (MCE) to be tested in CBA-N2a for giant clam fractions and the limit of quantification (LOQ) for CBA-N2a in our assays conditions were also assessed and were 3.10 ± 0.76 fg/µL, 10,869 pg/µL, and 0.014 ng P-CTX3C equiv./g of giant clam, respectively.

### 4.5. Liquid Chromatography Coupled with Tandem Mass Spectrometry (LC-MS/MS)

The procedure for liquid chromatography coupled with tandem mass spectrometry (LC-MS/MS) analyses was adapted from Yogi et al. (2011) [49]. Analyses were performed using an LC system (UFLC XR Nexera, SHIMADZU, Kyoto, Japan) coupled to a hybrid triple quadrupole-linear ion trap API4000 QTRAP mass spectrometer (SCIEX, Redwood City, CA, USA) equipped with a TurboV^®^ electrospray ionization source. A Zorbax Eclipse plus column (C_18_, 1.8 μm, 50 mm × 2.1 mm, Agilent technologies, Santa Clara, CA, USA) was employed at 40 °C and P-CTXs were eluted at 400 μL/min with a linear gradient using water as eluent A and methanol as eluent B, both eluents containing 2 mM ammonium formate and 50 mM formic acid. The elution gradient ran from 78 to 88% B over 10 min and was held for 4 min before returning to initial conditions and re-equilibration during 5 min. Five microliters of sample were injected onto the column. The instrument control, data processing and analysis were conducted using Analyst software. The API4000 QTRAP was operated in positive mode using Multi Reaction Monitoring (MRM). The pseudomolecular ions [M+NH_4_]^+^ and [M+H]^+^ were selected as precursor ions. The ions resulting in the successive losses of NH_4_ and/or water molecules were selected as product ions (see Roué et al. (2016) [17]). The MRM experiments were established by using the following source settings: curtain gas set at 25, ion spray at 5500 V, a turbogas temperature of 300 °C, gas 1 set at 40 and gas 2 set at 60 psi with an entrance potential of 10 V. Calibration was carried out using P-CTX3C standard purchased from Wako chemical. In addition, three P-CTX standards: P-CTX1B, P-CTX3B, and P-CTX3C, obtained from the Institut Louis Malardé’s bank of standards were injected to obtain a toxin profile for verification of retention times. The limit of detection (LOD) was statistically determined and found to be 0.66 ng P-CTX3C equiv./g of shellfish matrix.

## Figures and Tables

**Table 1:** Absolute toxicities and tissue distribution of toxins in giant clams exposed to *G. polynesiensis* cells (TB92 strain) based on CBA-N2a data. In each tank, each group of tissues was pooled from three giant clams.

Tank	Tissue	Absolute Toxicity of Tissue ^1^	Tissue Wet Weight [g] (Relative Weight Contribution, %)	Tissue Distribution of Toxins ^2^	Tissue Contribution to the Total Body Toxin Content
1	Viscera	12.2 ± 0.4	36 (26%)	3.2 ± 0.1	59%
	Flesh	3.0 ± 0.2	75 (54%)	1.6 ± 0.1	31%
	Mantle	2.6 ± 0.2	28.5 (20%)	0.5 ± 0.0	10%
2	Viscera	13.1 ± 0.8	37.5 (25%)	3.3 ± 0.2	71%
	Flesh	2.0 ± 0.2	72 (48%)	0.9 ± 0.1	20%
	Mantle	1.4 ± 0.2	39.5 (27%)	0.4 ± 0.1	9%
3	Viscera	13.6 ± 1.4	33.5 (26%)	3.5 ± 0.4	64%
	Flesh	3.0 ± 0.1	58.5 (45%)	1.4 ± 0.0	25%
	Mantle	2.1 ± 0.3	38 (29%)	0.6 ± 0.1	11%

^1^ The absolute toxicity of each tissue, expressed in ng P-CTX3C equiv./g tissue, was measured directly from the extracts of each tissue using the following formula: P-CTX3C EC_50_/sample EC_50_; ^2^ The tissue distribution of toxins, expressed in ng P-CTX3C equiv./g whole body, was calculated using the following formula: absolute toxicity × relative weight contribution.

**Table 2:** Toxin contents in contaminated giant clams after 0, 2, 4, and 6 days of detoxification, based on CBA-N2a data.

Days of Detoxification	Giant Clam Individual	Wet Weight [g]	Toxin Content ^1^
0	1	39.9	1.4 ± 0.2
	2	57.1	1.9 ± 0.1
	3	60.8	2.2 ± 0.4
	Average of 1–3	53 ± 11	1.8 ± 0.4
2	1	39.0	3.3 ± 0.4
	2	40.8	2.3 ± 0.1
	3	67.2	1.9 ± 0.4
	Average of 1–3	49 ± 16	2.5 ± 0.7
4	1	71.0	3.3 ± 0.4
	2	54.3	1.6 ± 0.2
	3	57.6	1.1 ± 0.2
	Average of 1–3	61 ± 9	2.0 ± 1.1
6	1	46.6	1.4 ± 0.1
	2	43.9	2.8 ± 0.8
	3	32.1	2.0 ± 0.3
	Average of 1–3	41 ± 8	2.1 ± 0.7

^1^ The toxin content, expressed in ng P-CTX3C equiv./g whole body, was measured directly from the extracts of each whole individual using the following formula: P-CTX3C EC_50_/sample EC_50_.
